# Triple-cation mixed-halide perovskites: towards efficient, annealing-free and air-stable solar cells enabled by Pb(SCN)_2_ additive

**DOI:** 10.1038/srep46193

**Published:** 2017-04-06

**Authors:** Yong Sun, Jiajun Peng, Yani Chen, Yingshan Yao, Ziqi Liang

**Affiliations:** 1 Department of Materials Science, Fudan University, Shanghai 200433, China

## Abstract

Organo-metal halide perovskites have suffered undesirably from structural and thermal instabilities. Moreover, thermal annealing is often indispensable to the crystallization of perovskites and removal of residual solvents, which is unsuitable for scalable fabrication of flexible solar modules. Herein, we demonstrate the non-thermal annealing fabrication of a novel type of air-stable triple-cation mixed-halide perovskites, FA_0.7_MA_0.2_Cs_0.1_Pb(I_5/6_Br_1/6_)_3_ (FMC) by incorporation of Pb(SCN)_2_ additive. It is found that adding Pb(SCN)_2_ functions the same as thermal annealing process by not only improving the crystallinity and optical absorption of perovskites, but also hindering the formation of morphological defects and non-radiative recombination. Furthermore, such Pb(SCN)_2_-treated FMC unannealed films present micrometer-sized crystal grains and remarkably high moisture stability. Planar solar cells built upon these unannealed films exhibit a high PCE of 14.09% with significantly suppressed hysteresis phenomenon compared to those of thermal annealing. The corresponding room-temperature fabricated flexible solar cell shows an impressive PCE of 10.55%. This work offers a new avenue to low-temperature fabrication of air-stable, flexible and high-efficiency perovskite solar cells.

Organo-metal halide perovskites have recently received exponentially increasing attention owing to their easy processability, low cost of materials, and excellent photovoltaic performance^1^,^2^,^3^. To date, the record power conversion efficiency (PCE) of perovskite based solar cells has reached ~22.1%^4^. The general chemical formula of perovskites is ABX_3_, where A is monovalent organic cation such as 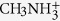 (MA^+^), 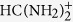 (FA^+^) and Cs^+^, B is divalent metal cation (e.g., Pb^2+^, Sn^2+^) and X is halide anion (e.g., Cl^−^, Br^−^, I^−^ or their mixtures)^5^. Among them, methylammonium lead triiodide (MAPbI_3_) is the most widely studied perovskite system, and has achieved PCEs up to 19.3%^6^. However, MAPbI_3_ is inevitably subjected to moisture, thermal and photo-instabilities, which largely restrict its future commercialization^7^,^8^. As an alternative, formamidinium lead triiodide (FAPbI_3_) has been recently developed because of its longer and broader light absorption along with better photo-stability than MAPbI_3_^9^. Yet the black perovskite-type trigonal structure (i.e., α-phase) of FAPbI_3_ readily transforms into the yellow non-perovskite hexagonal structure (i.e., δ-phase) at room temperature^10^. More recently, all-inorganic cesium lead triiodide (CsPbI_3_) perovskites have drawn intensive research interest for its superior thermal stability^11^. Unfortunately, its large band gap (~1.73 eV) is not suitable for PV applications and it is unstable in the photoactive α-phase in ambient atmosphere^12^.

To enhance the stability of neat perovskites, mixed cations and/or halides have been introduced into perovskite compounds. For instance, the incorporation of MAPbBr_3_ into FAPbI_3_ stabilized the perovskite phase of FAPbI_3_ and improved the solar cells up to >18% in PCE^13^. Very recently, by partial replacement of FA by Cs, the resulting FA_0.9_Cs_0.1_PbI_3_ exhibited significantly improved photo- and moisture stabilities^14^. Most recently, Grätzel *et al*. presented triple-cation type perovskites of Cs_x_(MA_0.17_FA_0.83_)_(100−x)_Pb(I_0.83_Br_0.17_)_3_ with high efficiency and more importantly, phase stability^15^. However, for all the above examples, thermal annealing is indispensable to crystallizing perovskite phases, which is highly unfavorable for constructing flexible solar cells on polyethylene terephthalate (PET) substrate.

We have previously manifested the non-thermal annealing fabrication of efficient planar MAPbI_3_ perovskite solar cells by inclusion of NH_4_Cl additive^16^. Furthermore, we have shown that the addition of Pb(SCN)_2_ to MAPbI_3_ led to increased crystal size, reduced traps and improved device stability^17^. Herein, we demonstrate such a type of air-stable triple-cation mixed-halide perovskites that exhibit highly efficient solar cell performance without the need of thermal annealing process, which is enabled by addition of Pb(SCN)_2_. By combination of FA, MA and Cs cations as well as mixed I and Br anions, the optimal FA_0.7_MA_0.2_Cs_0.1_Pb(I_5/6_Br_1/6_)_3_ (namely, FMC) is, by calculation, expected to form stable perovskite phases. By incorporation of Pb(SCN)_2_ additive, FA_0.7_MA_0.2_Cs_0.1_Pb(I_5/6_Br_1/6_)_3−x_(SCN)_x_ (donated as FMC-SCN) perovskite readily yields the increased crystal size and improved crystallinity in the absence of thermal annealing. Moreover, it aids to suppress the formation of morphological defects and PbI_2_ phase. As a result, a high PCE of 14.09% is obtained in such FMC perovskite based planar solar cells with alleviated hysteresis phenomenon.

## Results

Theoretical calculation was first used to acquire the best composition formula of stable perovskite phase. ABX_3_ perovskites with A = FA, MA or Cs, B = Pb, and X = I or Br compositions can adopt different crystal structures depending on the size and interaction of the A cation and the corner-sharing [BX_6_]^4^ octahedral^4^. Goldschmidt tolerance factor (*t*) is an empirical index for predicting stable crystal structures of perovskite materials, which can be calculated from the ionic radius of the atoms using equation (1)^18^.


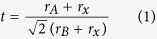


where *r*_A_, *r*_B_, and *r*_X_ are the effective ionic radii for A, B, and X ions, respectively. When the *t* value lies in the range of 0.8–1.06, cubic perovskite structures with high stability are formed^18^. Otherwise, non-perovskite structures may exist. In this work, we attempt to introduce an appropriate stoichiometric ratio of mixed MA, Cs, Br ions into typical FAPbI_3_ perovskite to obtain stable cubic-phases in the formula of FA_x_MA_y_Cs_1*−*x*−*y_Pb(I_z_Br_1*−*z_)_3_. The effective radius size of cation (*r*_cation_) and anion (*r*_anion_), effective tolerance factor (*t*_eff_) and octahedral factor (μ) can be estimated by equations (2,3,4,5)^18^:


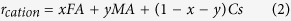



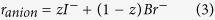



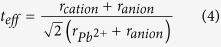



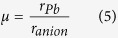


According to the previous reports, the ionic radii of FA^+^, MA^+^, Cs^+^, Pb^2+^, I^−^ and Br^−^ are calculated as 2.53, 2.16, 1.67, 1.02, 2.20 and 1.96 Å, respectively^19^,^20^. As a result, the optimal composition formula with high stability is FA_0.7_MA_0.2_Cs_0.1_Pb(I_5/6_Br_1/6_)_3_ (abbreviated as FMC) with a *t*_eff_ of 0.997 and μ of 0.47, which falls in the range of 0.8–1.06 and over 0.41, respectively^20^.

Next, we added a small amount of Pb(SCN)_2_ into FMC perovskite solution to investigate the effects on optical properties and crystalline morphology with/without thermal annealing. The one-step and ‘antisolvent’ methods were utilized to obtain FMC based perovskite films^21^. The precursor solution of FMC was prepared by dissolving a mixture of PbI_2_, FAI, MAI, CsI and PbBr_2_ in dimethylformamide (DMF) solvent with or without adding 5 wt% Pb(SCN)_2_. The mixed solution was then spin-coated on the substrate, immediately followed by exposure to toluene to induce the crystallization and form uniform films^21^. Thermal annealing was conducted at 100 °C for 10 min. Consequently, five sets of perovskite films were prepared including typical FAPbI_3_ (**A**), unannealed FMC (**B**), annealed FMC (**C**), unannealed FMC with Pb(SCN)_2_ (**D**), and annealed FMC with Pb(SCN)_2_ (**E**).

**Figure 1:**
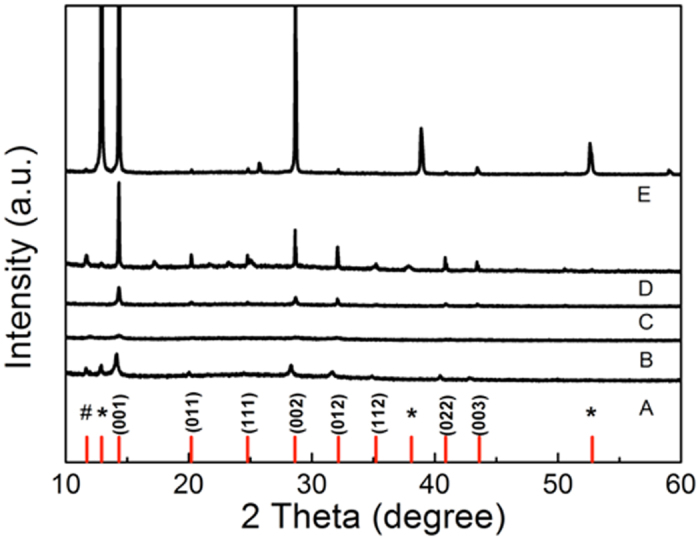
XRD patterns for different perovskite thin-films. Annealed FAPbI_3_ (A), unannealed FMC (B), annealed FMC (C), unannealed FMC with Pb(SCN)_2_ (D), and annealed FMC with Pb(SCN)_2_ (E). Note that the symbol ‘^#^’ and ‘*’ represents the δ-phase of FAPbI_3_ and the cubic PbI_2_, respectively.

X-ray diffraction (XRD) measurement was then employed to determine the roles of thermal annealing and Pb(SCN)_2_ additive on the formation of perovskite phases and crystallization in the above five samples. As shown in Fig. 1, all samples (**A**−**E**) exhibit two major peaks characteristic of perovskite at 14.34° and 28.61°, which are assigned to (001) and (002) crystal planes, respectively^22^. For typical FAPbI_3_ perovskite, two small peaks at 11.63° and 12.85° corresponding to the photo-inactive hexagonal δ-phase (i.e., yellow phase) of FAPbI_3_ and cubic PbI_2_, respectively, indicating the incomplete conversion of FAPbI_3_ into the photoactive black phase^15^.

By contrast, after introducing small amounts of MA, Cs, Br mixed ions in proportion to FMC, both peaks of yellow phase and PbI_2_ completely disappears, suggesting the complete conversion into photoactive black phase. In addition, all the peak intensities of annealed FMC are remarkably higher than those of unannealed FMC, which emphasizes the critical role of thermal annealing during the formation of perovskite phase.

Intriguingly, after the addition of Pb(SCN)_2_, even without thermal annealing, all the peaks are enhanced in intensity than those of annealed FMC. This suggests that the addition of Pb(SCN)_2_ additive can completely replace thermal annealing step to increase the crystallinity of perovskite phase. However, when the film of FMC with Pb(SCN)_2_ is annealed at 100 °C for 10 min, the peak intensities become tremendously high and the PbI_2_ phase unfortunately appear again, meaning that thermal annealing can further increase the perovskite crystallinity yet at the expense of largely decomposing the perovskite phases.

**Figure 2:**
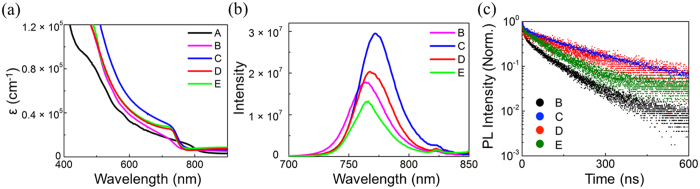
Optical characteristics of different perovskite thin-films. (**a**) UV-vis absorption coefficient (ε), (**b**) steady-state photoluminescence spectra and (C) time-resolved photoluminescence profiles of annealed FAPbI_3_ (A), unannealed FMC (B), annealed FMC (C), unannealed FMC with Pb(SCN)_2_ (D), and annealed FMC with Pb(SCN)_2_ (E).

Different crystallization dynamics of the above perovskites would strongly impact their optical properties. Thus, we measured and compared their optical absorption spectra. As shown in Fig. 2a, all FMC samples without or with Pb(SCN)_2_ exhibit notably stronger absorption at 400–750 nm than FAPbI_3_ counterpart. Meanwhile, the absorption intensity of annealed FMC sample is considerably larger than that of unannealed analogue, indicating the important role of thermal annealing process in the formation of perovskite phase. However, upon adding Pb(SCN)_2_, both unannealed and annealed FMC samples show almost identical absorption spectra, meaning that perovskites phase can be successfully formed even at room temperature with the aid of Pb(SCN)_2_.

In addition to crystalline structures, morphological defects or traps in perovskite films would also influence optical properties. Therefore, the steady-state photoluminescence (PL) spectra of different FMC films without or with Pb(SCN)_2_ were characterized. As shown in Fig. 2b, the PL intensity of annealed FMC samples is larger than that of unannealed ones, implying the decrease of defects under thermal annealing^23^. In contrast, the PL intensity of unannealed FMC is adversely higher than that of annealed one after the addition of Pb(SCN)_2_, indicating that more defects are formed during thermal annealing process. Time-resolved PL (TRPL) was further measured to examine the recombination dynamics of the photo-excited states in different FMC films. Note that the TRPL measurement was performed at 760 nm using a 485 nm incident laser, and the analysis of biexponetial fit parameters for FMC based perovskites. The PL kinetics data are summarized in Supplementary Table S1. As shown in Fig. 2c, thermal annealing and addition of Pb(SCN)_2_ can both remarkably prolong the PL lifetime of FMC perovskites from 53.82 ns to 178.23 ns and 117.25 ns, respectively. However, after the perovskite of FMC with Pb(SCN)_2_ is thermally annealed, the PL lifetime is only slightly increased to 71.97 ns. Therefore, both PL and TRPL results indicate that adding Pb(SCN)_2_ as well as thermal annealing allows to significantly reduce crystal defects and increase the PL lifetime, resulting in suppressed non-radiative recombination and improved charge transport^24^.

**Figure 3:**
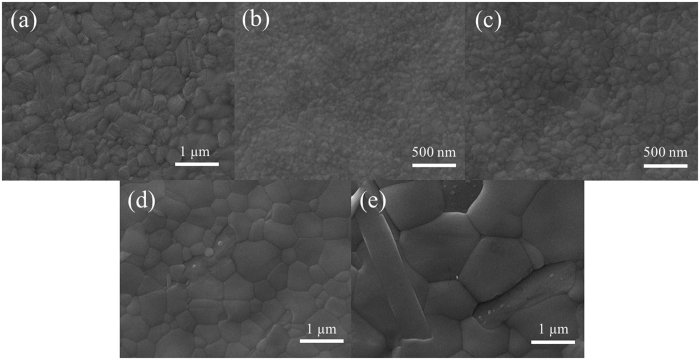
FE-SEM images of different perovskite thin-films. (**a**) Annealed FAPbI_3_ film, (**b**) unannealed and (**c**) annealed FMC films, (**d**) unannealed and (**e**) annealed FMC films with Pb(SCN)_2_.

Field-emission scanning electron microscopy (FE-SEM) was used to further verify the different roles of thermal annealing process and Pb(SCN)_2_ additive on the film morphology and crystallization of FMC based perovskites. As shown in Fig. 3a, annealed FAPbI_3_ film shows a rough surface consisting of numerous packed crystals. By contrast, smoother surface can be all obtained in the FMC based films (Fig. 3b–e). Moreover, by comparing Fig. 3b,c, thermal annealing can slightly increase the crystal size of FMC. Upon the addition of Pb(SCN)_2_ and without thermal annealing, the crystal size significantly increases up to 1 *μ*m, suggesting that Pb(SCN)_2_ additive results in enhanced crystallinity at room temperature (Fig. 3d). This is completely consistent with the XRD and PL results and also in good accordance with our previous report^17^. However, as shown in Fig. 3e, when the film of FMC with Pb(SCN)_2_ is thermally annealed at 100 °C for 10 min, the crystal size approaches 2 *μ*m while rod-like PbI_2_ crystals are also formed and generate more morphological defects, which will severely undermine the charge transport and device performance of perovskite solar cells. Moreover, energy dispersive X-ray (EDX) analysis (Supplementary Figure S1) shows that the atomic weight of sulfur element in FMC with Pb(SCN)_2_ is slightly decreased after thermal annealing, indicating that most of SCN^−^ remains, which is in accordance with previous report^17^,^25^,^26^,^27^.

We then evaluated the moisture stability of FMC based perovskites. All the typical FAPbI_3_ and FMC based perovskites were first stored in air with high relative humidity (RH) around 80% at 20 °C. As shown in Supplementary Figure S2, all the films exhibit black and smooth surface in the beginning. When the exposure time is increased to 2 h, a large area of FAPbI_3_ (**A**) quickly turns yellow while FMC perovskites (**B**,**C**) preserve blackish only with tiny white spots. It means the improved stability of FMC than FAPbI_3_, which is in accordance with the calculation of tolerance factor. By contrast, Pb(SCN)_2_ treated FMC perovskite films turn slightly into brownish without tiny spots during the exposure, indicating that the stability of FMC is largely preserved after the addition of Pb(SCN)_2_. However, all the above films degrade too fast to trace the degradation trend under high RH. Therefore, their degradation mechanism under modest moisture—with RH of 65% at 20 °C—was carefully examined by optical absorption studies. As shown in Supplementary Figure S3a, the absorption curve of FAPbI_3_ immediately becomes flat after 120 min’s exposure in air. By contrast, the absorption intensities of annealed FMC only slightly decrease after 1440 min while the unannealed FMC without Pb(SCN)_2_ first slightly degrades after 300 min and then rapidly decomposes after 1080 min (Figure S3b,c), suggesting that remarkably improved moisture stability is achieved in FMC based films.

**Figure 4:**
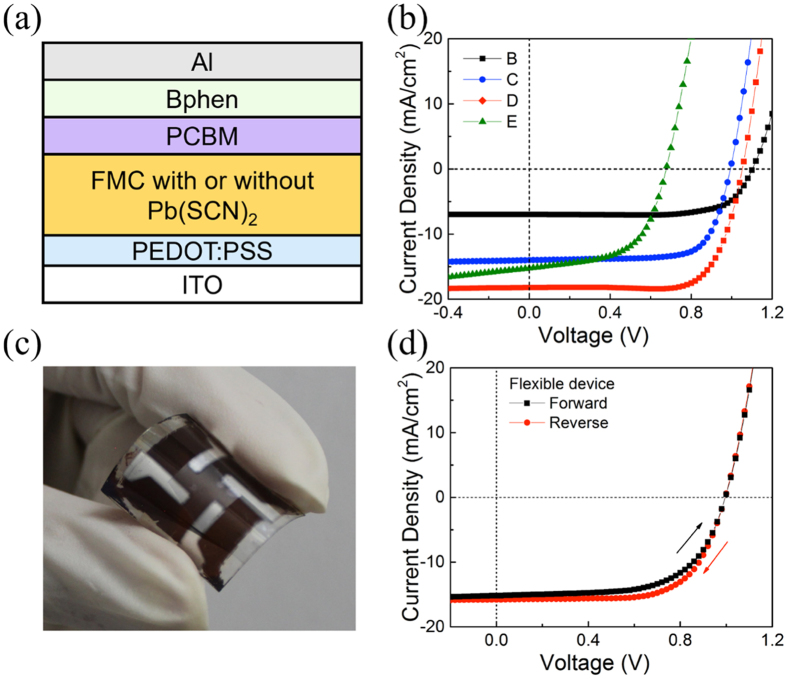
Schematic of the device architecture, the photovoltaic performances of perovskite solar cells and the image of a flexible cell. (**a**) Schematic configuration of FMC perovskites based bilayered solar cells, and (**b**) their representative photocurrent density−voltage (*J* − *V*) characteristics of unannealed FMC (B), annealed FMC (C), unannealed FMC with Pb(SCN)_2_ (D), and annealed FMC with Pb(SCN)_2_ (E) under light irradiation of 100 mW/cm^2^ at reverse scan. (C) Photograph and (D) *J* − *V* characteristics of D based flexible solar cell under reverse and forward scans.

Next, we investigated the effects of thermal annealing and addition of Pb(SCN)_2_ on the FMC perovskites based planar solar cells of ITO/PEDOT:PSS/perovskite/PC_61_BM/Bphen/Al, as schematically depicted in Fig. 4a. Figure 4b presents the photocurrent density–voltage (*J* − *V*) curves of the optimal devices with reverse scan under simulated AM 1.5 G sunlight irradiation. The complete *J* − *V* profiles with both reverse and forward scan directions are shown in Supplementary Figure S4, and the photovoltaic parameters are summarized in Table 1. In the FMC systems, thermal annealing assists to greatly increase the short-circuit current density (*J*_SC_) from 6.98 to 14.01 mA/cm^2^ at reverse scan with identical open-circuit voltage (*V*_OC_) of ~1.05 V and fill factor (FF) of ~72%, which coincides well with the optical absorption results, and thus doubles the PCE from 5.52% to 10.15%. However, hysteresis phenomenon still obviously exists in the annealed FMC cells as indicated in Figure S4b.

On the contrary, after adding Pb(SCN)_2_ and without thermal annealing, a significantly higher *J*_SC_ of 18.21 mA/cm^2^ is obtained while preserving about the same *V*_OC_ and FF, resulting in the highest PCE of 14.09% at reverse scan. Meanwhile, the hysteresis phenomenon is greatly suppressed in the unannealed device of FMC with Pb(SCN)_2_. These results explicitly suggest that the addition of Pb(SCN)_2_ is remarkably more effective in improving not only crystalline morphology but also device performance than thermal annealing in FMC based solar cells. Supplementary Figure S5 displays the efficiency histograms of 10 individual unannealed devices of FMC with Pb(SCN)_2_ at reverse scans, which indicates the good reproducibility with an average PCE of 11.97%. Moreover, such low-temperature processing will greatly accelerate the large-scale production of flexible perovskite solar modules. As a demonstration, we therefore fabricated such a flexible solar cell of FMC with Pb(SCN)2 on PET substrate at room temperature (Fig. 4c), which exhibits impressive PCEs of 10.55% and 9.48% under reverse and forward scans, respectively, with little hysteresis (Fig. 4d).

However, when the device of FMC with Pb(SCN)_2_ is thermally annealed at 100 °C for 10 min, *J*_SC_ is slightly reduced to 15.24 mA/cm^2^ at reverse scan while *V*_OC_ and FF are dramatically decreased to 0.68 V and 57.05%, respectively, resulting a much lower PCE of 5.91%. This agrees well with the obtained results of almost unchanged optical absorption yet significantly increased defects, the latter of which is indicated from PL and SEM characterizations and originate from the largely deteriorated perovskite crystalline morphology and the formation of PbI_2_ phase.

Lastly, we evaluated thermal stabilities of FMC with Pb(SCN)_2_ and their solar cells. The thermal gravimetric analysis (TGA) profiles (Supplementary Figure S6) show that FMC displays a degradation temperature onset at 200 °C and remains unchanged after the addition of Pb(SCN)_2_. Furthermore, we investigated the effect of modest thermal annealing on the cell performance. Upon adding Pb(SCN)_2_ and thermal annealing at 50 °C for 5 and 10 min, the PCE is only slightly decreased from 14.09% to 11.61% and 12.14%, respectively, at reverse scan (Supplementary Figure S7 and Table S2). These results imply that the FMC with Pb(SCN)_2_ based devices exhibit the same good thermal stability as those of FMC.

## Discussion

In summary, we have shown a novel type of air-stable perovskites, FA_0.7_MA_0.2_Cs_0.1_Pb(I_5/6_Br_1/6_)_3_ where the influences of thermal annealing process and adding Pb(SCN)_2_ are comparatively studied. Both of them contribute equally to not only increasing the crystallinity, optical absorption and PL lifetime of perovskites, but also hindering the formation of morphological defects and PbI_2_ phase, and suppressing charge recombination. In FMC perovskites based planar solar cells, Pb(SCN)_2_ additive is found to enhance the device performance (in particular *J*_SC_) and suppress the hysteresis phenomenon more significantly than thermal annealing, yielding the best PCE of 14.09%. As such, the flexible solar cell based on unannealed FMC-SCN exhibits an impressive PCE of 10.55%. This work offers a new yet scalable avenue towards fabricating efficient perovskite solar cells at room temperature with good moisture and thermal stabilities.

## Methods

### Chemicals

All chemicals were purchased from J&K Scientific, Ltd. (China) and Acros Organics unless indicated, and used as received. The phenyl-C61-butyric acid methyl ester (PC_61_BM) was obtained from Nano-C, Inc.

### Fabrication of perovskite films

The one-step and ‘antisolvent’ methods were utilized to obtain FAPbI_3_ and FMC based films. FAPbI_3_ perovskite solution was prepared by dissolving PbI_2_ (0.323 g), and FAI (0.120 g) in DMF. The precursor solution of FMC was prepared by dissolving a mixture of PbI_2_ (0.346 g), FAI (0.121 g), MAI (0.032 g), CsI (0.026 g) and PbBr_2_ (0.092 g) in 1 mL of dimethylformamide (DMF) solvent with or without adding 5 wt% Pb(SCN)_2_. The mixed solution was then spin-coated at 4000 rpm for 30 s on the substrate. After a specific delay time (e.g., 6 s) during spin-coating, a second solvent of toluene was immediately added to the substrate. Thermal annealing of FMC and FAPbI_3_ was conducted at 100 °C and 175 °C for 10 min, respectively.

### Characterization

Optical absorption and photoluminescence spectra of samples were acquired on Agilent 8453 UV-Visible spectrophotometer and a Horiba FluoroMax^®^-4 spectrofluorometer, respectively. X-ray diffraction pattern data for 2θ values were collected with a Bruker AX D8 Advance diffractometer with nickel filtered Cu Kα radiation (λ = 1.5406 Å). Field-emission scanning electron microscopy images coupled with energy-dispersive X-ray elemental analysis were acquired on Philips XL-30 field-emission gun at an accelerating voltage of up to 30 kV. Fluorescence lifetimes were measured using FluoroLog-3 modular spectrofluorometer (HORIBA Scientific, Inc.) at 760 nm using a 485-nm incident laser. Thermogravimetry analysis was performed on a TA Q500 at a heating rate of 10 °C/min from 25 to 600 °C under nitrogen atmosphere.

### Device and measurements

Patterned indium tin oxide (ITO) on glass and polyethylene terephthalate (PET) substrates (12 Ω, Thin Film Devices, Inc.) were cleaned sequentially in an ultrasonic solvent bath of deionized water, acetone, and isopropyl alcohol. After drying with a stream of nitrogen, the substrates were treated in a UV Ozone (UVO) cleaner for 20 min. For the regular cells, a thin layer (~30 nm) of PEDOT:PSS (CleviosTM P VP AI 4083, Heraeus) was spin-coated onto the ITO at 5000 rpm for 60 s and then baked in air at 130 °C for 20 min. The PEDOT:PSS-coated substrates were immediately transferred to a N_2_-filled glovebox for making the active layer. After the formation of the perovskite layer, a second layer of PC_61_BM was spin-coated from chlorobenzene solution (20 mg/mL) at 3000 rpm for 30 s. The samples were then loaded into a glovebox-integrated deposition chamber and pumped down to a pressure of <10^−5^ Pa. A sequence of Bphen (3 nm) and Al (80 nm) layers was deposited by thermal evaporation through a shadow mask at a rate of 1.3 and 1 Å/s, respectively. The active area as defined shadow mask is ~0.04 cm^2^. The sample was mounted inside a nitrogen-filled sample holder with a quartz optical window for subsequent measurements.

The light *J* − *V* curves were measured on a Keithley 2400 source meter unit under AM 1.5 G light illumination with a Newport-Oriel (Sol3A Class AAA Solar Simulator, 94043 A) solar simulator operating at an intensity of 100 mW cm^−2^. The light intensity was calibrated by a certified Oriel reference cell (91150 V) and verified with a NREL calibrated, filtered silicon diode (Hamamatsu, S1787–04). The *J* − *V* profiles were obtained under both forward (−0.5 V → +1.5 V) or reverse (+1.5 V → −0.5 V) scans at a rate of 10 mV/s.

## Additional Information

**How to cite this article**: Sun, Y. *et al*. Triple-cation mixed-halide perovskites: Towards efficient, annealing-free and air-stable solar cells enabled by Pb(SCN)_2_ additive. *Sci. Rep.* **7**, 46193; doi: 10.1038/srep46193 (2017).

**Publisher's note:** Springer Nature remains neutral with regard to jurisdictional claims in published maps and institutional affiliations.

## Supplementary Material

Supplementary Information

## Figures and Tables

**Table 1:** Photovoltaic parameters of FMC based solar cells.

Sample	Thermal annealing	Scanning direction	PCE (%)	*J*_SC_ (mA/cm^2^)	*V*_OC_ (V)	FF (%)
FMC	×	Reverse	5.52	6.98	1.10	71.87
		Forward	5.17	9.11	1.08	52.59
	√	Reverse	10.15	14.01	1.00	72.49
		Forward	9.51	16.95	0.94	59.67
FMC + Pb(SCN)_2_	×	Reverse	14.09	18.21	1.06	72.97
		Forward	12.71	18.05	1.04	67.70
	√	Reverse	5.91	15.24	0.68	57.05
		Forward	3.96	13.84	0.62	46.19
Flexible	×	Reverse	10.55	15.80	1.00	66.76
		Forward	9.48	15.17	1.00	62.49
