# Supplementary material for: Triple-cation mixed-halide perovskites: towards efficient, annealing-free and air-stable solar cells enabled by Pb(SCN)2 additive
Source: Sci Rep. 2017 Apr 6;7:46193. doi: 10.1038/srep46193 (PMC5382775; doi:10.1038/srep46193)
Supplement: Supplementary Information [file srep46193-s1.pdf]

Supplementary information

Triple-cation mixed-halide perovskites: towards efficient, annealing-free and air-stable solar cells enabled by $Pb(SCN)_2$ additive

Yong Sun, Jiajun Peng, Yani Chen, Yingshan Yao and Ziqi Liang*

Department of Materials Science, Fudan University, Shanghai 200433, China

* zqliang@fudan.edu.cn

Sample	Thermal Annealing	τ_1 (ns)	b1 (%)	τ_2 (ns)	b2 (%)	Average τ (ns)	CHISQ
FMC	×	8.98	8.71	102.87	91.29	53.82	1.25
	√	47.90	9.71	251.96	90.29	178.23	1.17
FMC + $Pb(SCN)_2$	×	4.74	1.52	185.08	98.48	117.25	1.14
	√	98.46	98.00	5.08	2.00	71.97	1.07

Table S1. Analysis of biexponetial fit parameters for PL kinetics data of FMC perovskites

* Note that when CHISQ approaches 1, the fitting results are closer to actual data.

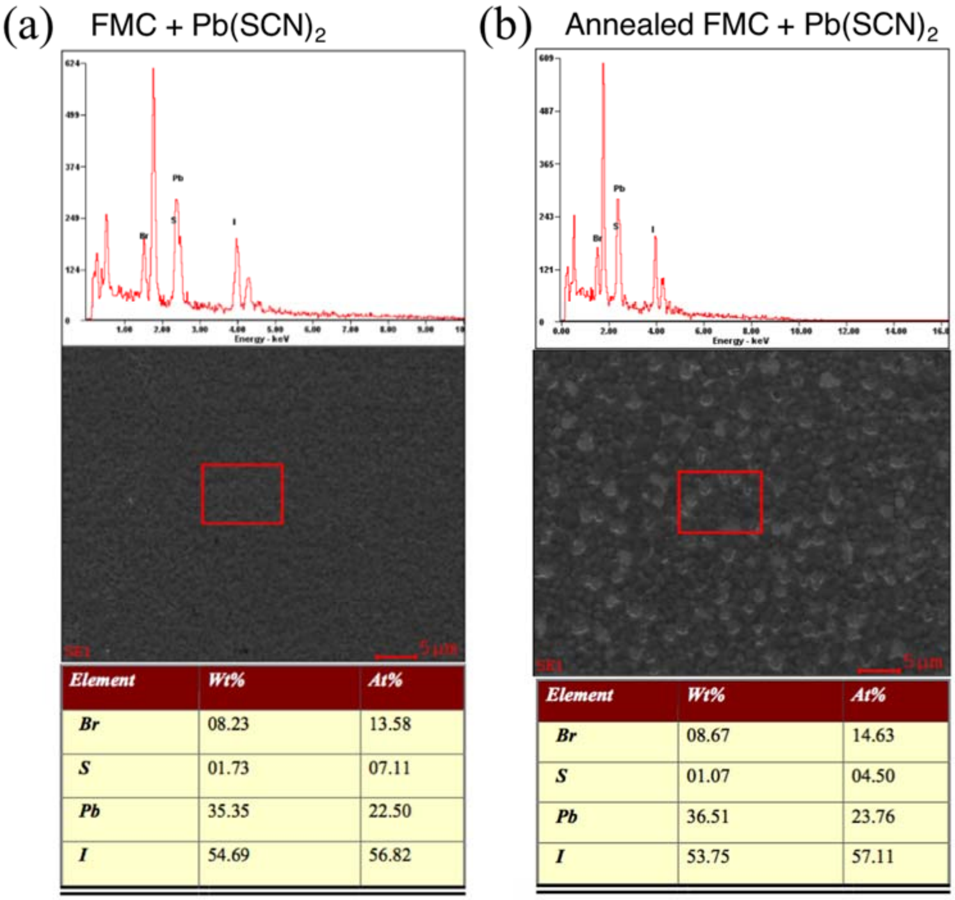

Figure S1. Energy dispersive X-ray (EDX) analysis of (a) unannealed and (b) annealed FMC with $Pb(SCN)_2$ films.

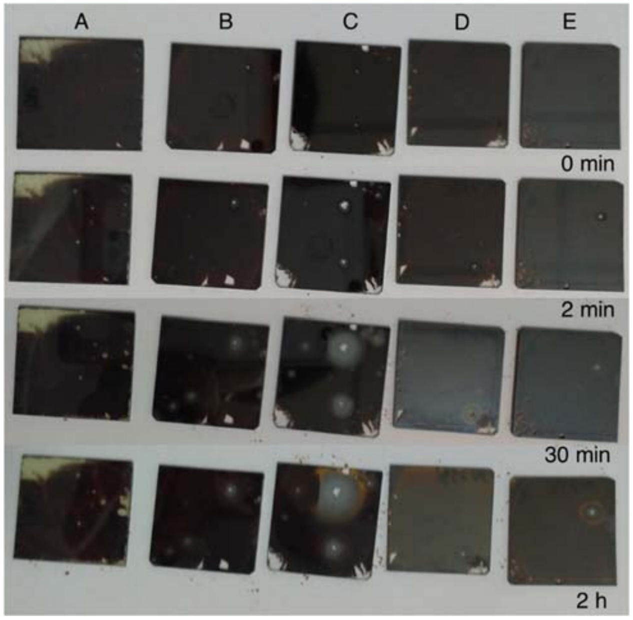

Figure S2. Photographs of annealed $FAPbI_3$ (**A**), unannealed FMC (**B**), annealed FMC (**C**), unannealed FMC with $Pb(SCN)_2$ (**D**), and annealed FMC with $Pb(SCN)_2$ (**E**) thin films on glass substrate over time, which are stored in air at 20 °C with a high humidity of 80%.

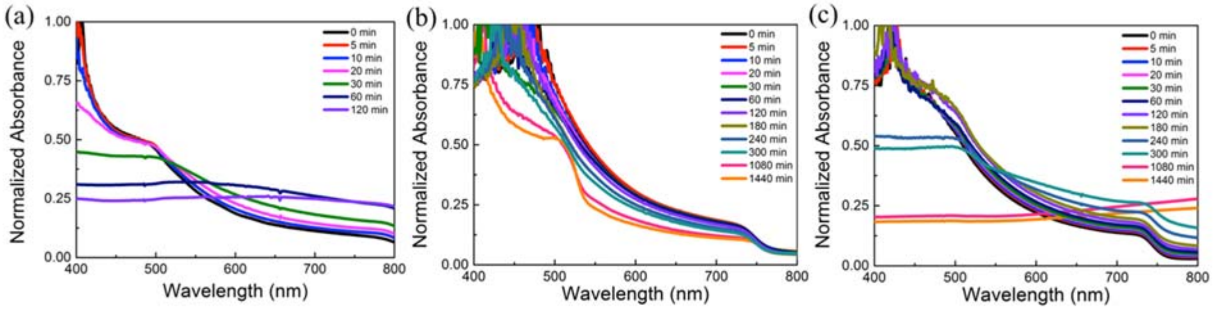

Figure S3. Optical absorption spectra of (a) annealed $FAPbI_3$, (b) annealed FMC and (c) unannealed FMC with $Pb(SCN)_2$ over time when they are stored in air at 20 °C with a modest humidity of 65%.

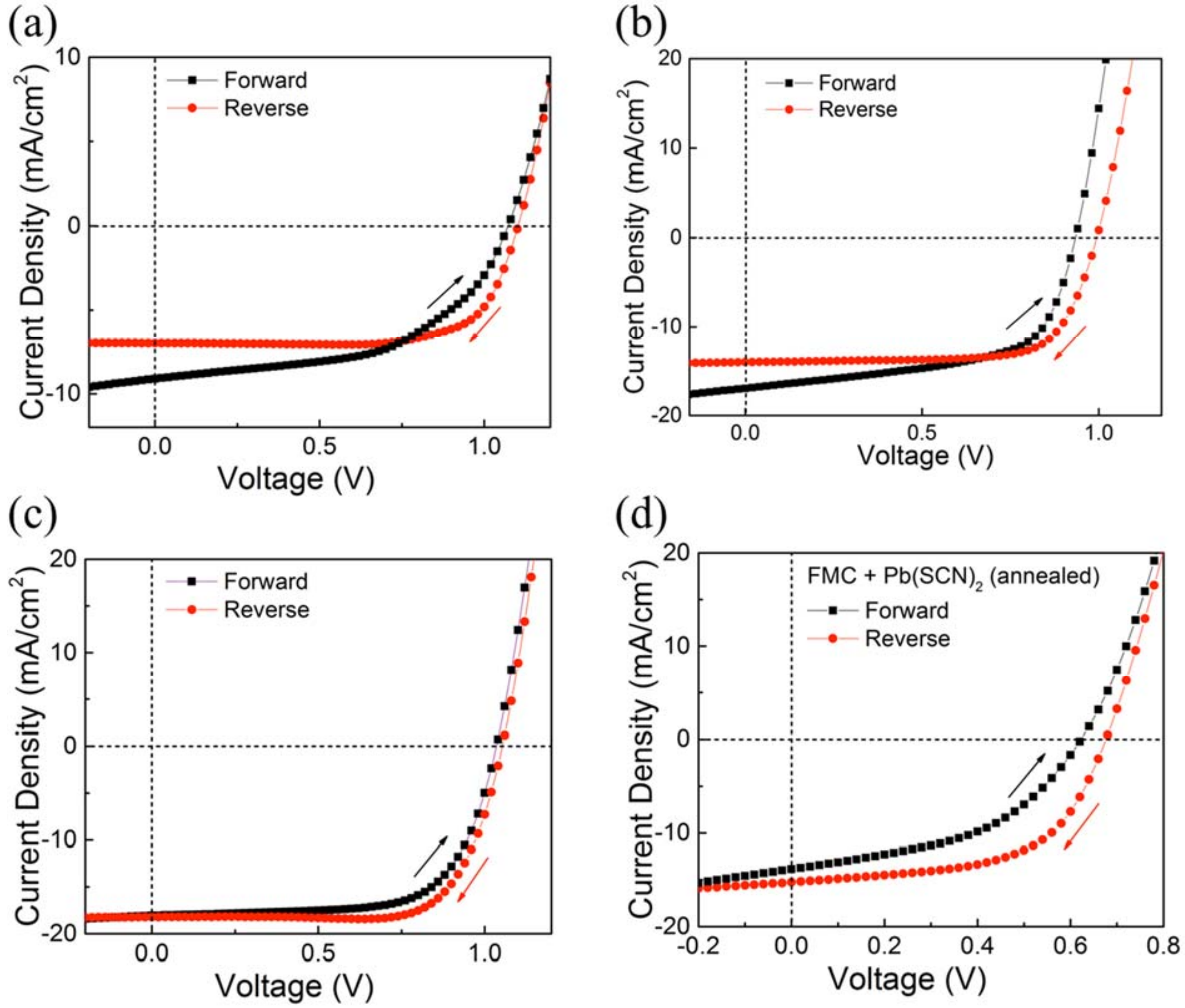

Figure S4. Representative current density−voltage (*J–V*) characteristics of (a) unannealed FMC, (b) annealed FMC, (c) unannealed FMC with $Pb(SCN)_2$, and (d) annealed FMC with $Pb(SCN)_2$ based planar solar cells under AM 1.5G simulated light at forward and reverse scans, respectively.

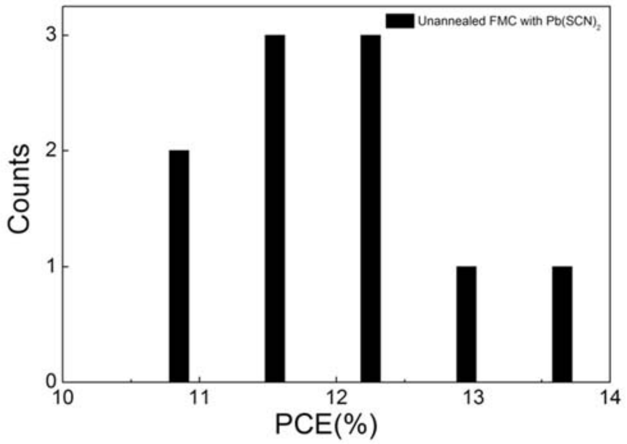

Figure S5. Power conversion efficiency (PCE) histograms of unannealed FMC with $Pb(SCN)_2$ based planar solar cells measured at reverse scan.

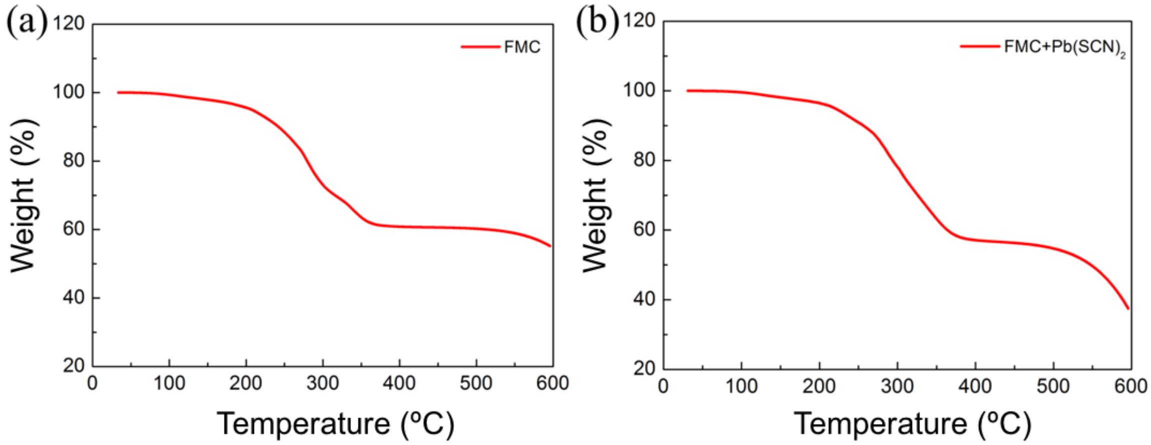

Figure S6. TGA curves of (a) FMC and (b) FMC with $Pb(SCN)_2$.

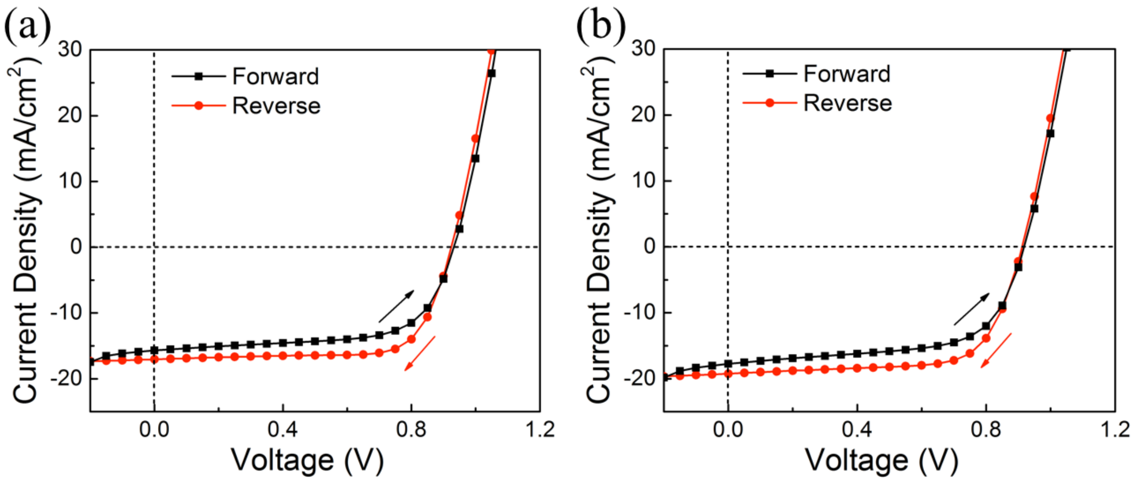

Figure S7. Representative current density−voltage (*J–V*) characteristics of annealed FMC with $Pb(SCN)_2$ at 50 ºC for (a) 5 min, and (b) 10 min based planar solar cells under AM 1.5G simulated light at forward and reverse scans, respectively.

Sample	Thermal annealing	Scanning direction	PCE (%)	J_{SC} (mA/cm²)	V_{OC} (V)	FF (%)
FMC + $Pb(SCN)_2$	50 ºC, 5 min	Reverse	11.61	17.05	0.90	75.67
		Forward	9.53	15.69	0.95	63.89
	50 ºC, 10 min	Reverse	12.14	19.24	0.90	70.08
		Forward	10.21	17.75	0.90	63.90

Table S2. Summary of photovoltaic parameters of annealed FMC with $Pb(SCN)_2$ based cells
